# PReS-FINAL-2207: Results from a multicenter international registry of Familial Mediterranean Fever: validation of the new set of pediatric diagnostic criteria

**DOI:** 10.1186/1546-0096-11-S2-P197

**Published:** 2013-12-05

**Authors:** E Demirkaya, S Ozen, C Saglam, T Turker, A Duzova, P Woo, I Konè-Paut, M Doglio, G Amarian, J Frenkel, Y Uziel, A Insalaco, L Cantarini, M Hofer, S Boiu, C Modesto, A Bryant, D Rigante, E Papadopoulou-Alataki, S Guillaume-Czitrom, N Ruperto, M Gattorno

**Affiliations:** 1 FMF Arthritis Vasculitis and Orphan Disease Research in Paediatric Rheumatology (FAVOR), Turkey; 2 Pediatric Rheumatology, Pediatric Rheumatology, Hacettepe University, School of Medicine, Ankara, Turkey; 3 Epidemiology, Gulhane Military Medical Faculty, Ankara, Turkey; 4 Rheumatology, Istanbul University, Istanbul, Turkey; 5 Pediatric Rheumatology, UCL, London, UK; 6 Pediatric Rheumatology, University of Paris SUD, Paris, France; 7 Pediatric Rheumatology, Ospedale Gaslini, Genoa, Italy; 8 National Pediatric Familial Mediterranean Fever Centre, Institute of Child and Adolescent Health, Yerevan, Armenia; 9 Pediatrics, University Medical Center Utrecht, Utrecht, The Netherlands; 10 Pediatrics, Meir Medical Centre, Kfar Saba, Israel; 11 Reumatologia, Ospedale Pediatrico Bambin Gesù, Rome, Italy; 12 Rheumatology, Policlinico le Scotte, University of Siena, Siena, Italy; 13 Pediatric Rheumatology, Centre Hospitalier Universitaire Vaudois, Lausanne, Switzerland; 14 Pediatric Rheumatology, Université Paris-Descartes, Paris, France; 15 Reumatologia, Hospital Valle de Hebron, Barcelona, Spain; 16 Pediatrics, Università Cattolica Sacro Cuore, Rome, Italy; 17 Fourth Department of Pediatrics, Aristotle University of Thessaloniki Papageorgiou Hospital, Thessaloniki, Greece; 18 Pediatric Rheumatology, Bicêtre-hôpitaux universitaires Paris-Sud, Bicetre, France

## Introduction

FMF diagnosis is made clinically and may be supported by identifying mutations in the MEFV gene. The most commonly used diagnostic criteria for FMF are those of Tel Hashomer and the Livneh criteria, which have been established in the Jewish adult population. Recently, a Turkish group (Yalçınkaya-Özen) proposed new criteria for diagnosis of FMF in children.

## Objectives

We aimed to analyze the validity of the Turkish diagnostic criteria for pediatric FMF in a large international registry.

## Methods

The study group is consisted of 339 FMF patients diagnosed according to Tel Hashomer criteria. A control group of 377 patients were diagnosed with other periodic fever syndromes including MKD, TRAPS, CAPS, PFAPA and undefined periodic fever. Both groups were evaluated according to the Tel Hashomer, Livneh criteria and the new set of pediatric diagnostic criteria.

## Results

The sensitivity and specificity of the Tel Hashomer criteriaand Livneh criteria in our study were 35.1% and 97.7%, 77.6% and 45.9%, respectively. The presence of two or more of these new five criteria diagnosed FMF with a high sensitivity of 87.4% and a negative predictive value (NPV) of 74.8%. When we used at least three pediatric criteria, the discrimination of the diseases other than FMF reached the highest specificity of 88.2% and the positive predictive value (PPV) of 82.9% at the expense of sensitivity. If all the new sets of criteria were met, the specificity and sensitivity were 99.6% and 5.6%, respectively with a PPV of 94.1% and an NPV of 49.2%. Our study showed that ethnicity had no impact on the validation.

## Conclusion

The Tel Hashomer diagnostic criteria were found to have high specificity (97.7%) for the diagnosis of FMF, whereas pediatric criteria had a higher sensitivity (87.4%) if at least two out of its five criteria were met. The small number of patients with amyloidosis or erysipelas like erythema and the response to colchicine therapy constituted the drawbacks in assessing the patients with Tel Hashomer criteria. The Livneh criteria were also found to have high specificity (45.9%) for the discrimination of the diseases other than FMF, whereas pediatric criteria were more precious than the Livneh criteria because of that it's higher sensitivity rate for FMF diagnosis in pediatric patients. Our analysis showed that the pediatric criteria had performed better in diagnosing patients with FMF in childhood in respect to Tel Hashomer and the Livneh criteria.

## Disclosure of interest

None declared.

